# Middle-aged Korean women’s experiences of physical activity during the transition to menopause: a grounded theory approach

**DOI:** 10.4069/whn.2025.08.25

**Published:** 2025-09-30

**Authors:** Hee Jung Cho, Sukhee Ahn

**Affiliations:** 1 College of Nursing, Chungnam National University, Daejeon, Korea; 2 College of Nursing, Sungshin Women’s University, Seoul, Korea

**Keywords:** Exercise, Menopause, Middle aged, Qualitative research, Women

## Abstract

**Purpose:**

This study aimed to comprehensively explain middle-aged Korean women’s experiences of physical activity during the transition to menopause.

**Methods:**

The participants were 13 middle-aged women in the menopausal transition who engaged in physical activities more than three times per week for at least 12 weeks. Data were collected through in-depth interviews and participatory observations from September 2022 to June 2023 and analyzed using Strauss and Corbin’s grounded theory methods.

**Results:**

The core category identified was “continuing my new daily life while improving my body and mind.” The central phenomenon was “desperation for change,” with causal conditions including “inconvenience with menopausal symptoms” and “physical and mental burnout.” The contextual condition was “surviving,” while the action/interaction strategies were “finding the right physical activity,” “focusing on physical activity,” “enjoying physical activity,” “practicing in life,” and “continuing new challenges.” Intervening conditions included “recognizing the need for physical activity” and “doing it together.” Consequences were identified as “settling into a normal life” and “moving forward.”

**Conclusion:**

This study demonstrates that regular physical activity supports a healthy menopause transition by enhancing both physical and mental well-being, thereby enabling women to adapt in positive directions through life changes. When women earnestly seek change, maintain an active attitude toward life, recognize the importance of physical activity, and utilize support systems, they continue to grow and successfully navigate the transition to menopause.

## Introduction

During the transition to menopause, women undergo diverse physical and psychological changes, including menopausal symptoms that negatively affect overall daily life. Common symptoms during this transition include vasomotor disturbances, sleep disorders, depression, anxiety, and cognitive decline. In addition, gradual decreases are observed in cardiovascular and cardiometabolic health, bone integrity, and physical performance [1]. Consequently, women become increasingly vulnerable to disease during this period, and negative menopausal experiences may be perceived as a serious crisis or major life problem [2].

The menopausal transition can be defined as a natural adaptive process, shifting from the premenopausal to the postmenopausal stage of life [3]. A healthy transition generally promotes stability, whereas an unhealthy transition may lead to a comprehensive crisis of physical and psychological health, necessitating the adoption of appropriate strategies for adaptation [4]. The most characteristic changes during this period are menopause-related symptoms. Most women experience one or more such symptoms, which not only diminish quality of life [5] but also heighten anxiety about aging [6]. Estrogen deficiency further contributes to physical changes, including loss of muscle mass and strength [7], increases in body fat, decreases in lean body mass [8], and elevated risks of dyslipidemia [9]. Above all, these combined changes, occurring alongside the aging process, significantly undermine the quality of life for women undergoing the menopausal transition.

Therefore, nursing approaches are required to improve the quality of life of women undergoing the menopausal transition and to help them cope with various negative changes [10,11]. Physical activity can be recommended as a practical strategy to foster positive changes in their lives. Conversely, insufficient physical activity during this transition increases the prevalence of metabolic syndrome [12], negatively influencing overall health. In contrast, regular physical activity not only alleviates menopausal symptoms but also enhances both physical and mental health. Accordingly, it can be applied as a major practical strategy for women in the menopausal transition [13,14]. In particular, women often become acutely aware of rapid bodily changes during menopause, and engaging in physical activity at this time can influence their subjective perception of health while also producing psychological benefits simply through the attempt to participate [15]. Moreover, regular physical activity is effective for weight control [16], prevention of bone mineral density loss [17], and maintenance of skeletal muscle mass [18]. It also exerts a positive effect on the regulation of estrogen and depression-related hormones [19], thus offering significant benefits in overcoming diverse transitional challenges faced by menopausal women.

However, most quantitative studies on physical activity have primarily focused on its impact and effects on the physical and mental health of menopausal women, which limits a deeper understanding of participants’ lived experiences. Qualitative studies, in contrast, have reported experiences such as overcoming bodily changes characteristic of midlife through physical activity [20], seeking meaning and identity in life [21], recognizing the moderating effects of physical activity on menopausal symptoms during the transition [22], and engaging in activity to manage these symptoms [23]. Nevertheless, few studies have concentrated on the transitional process as it unfolds over time.

Therefore, this study applies grounded theory to explore and understand, from the perspectives of middle-aged women actually undergoing the menopausal transition, how they interact within specific contextual circumstances and adapt to life changes through regular physical activity. The research question guiding this investigation is: “What are the physical activity experiences of middle-aged women undergoing the menopausal transition?” The grounded theory approach is suitable for this study because it derives theoretical insights from participants’ experiences, thereby providing a comprehensive understanding and explanation of the phenomenon [24,25]. Through this, the study aims to provide foundational data for developing nursing strategies to support middle-aged women in adapting to the menopausal transition in a healthy direction.

## Methods

**Ethics statement**: This study was approved by the Institutional Review Board of Chungnam National University (IRB-202205-SB-057-01). Informed consent was obtained from the participants.

### Study design

This study is qualitative research applying Strauss and Corbin’s [25] grounded theory approach to deeply explore and describe the process of physical activity experiences among middle-aged women undergoing the menopausal transition. In addition, the study was described in accordance with the SRQR (Standards for Reporting Qualitative Research) guidelines [26].

### Study participants

In this study, the participants were middle-aged women experiencing menopausal symptoms, the most characteristic phenomena during the menopausal transition. Purposeful sampling was applied to recruit participants suitable for the research aim. The specific inclusion criteria were women aged 45–60 years who recognized discomfort caused by one or more menopausal symptoms and who had consistently engaged in regular exercise for at least 12 weeks, with a minimum frequency of twice a week and at least 50 minutes per session. Women currently receiving hormone therapy were excluded in order to focus solely on physical activity experiences. Recruitment was conducted among members enrolled in the group exercise program at J Fitness Center in Seoul, following the guidance of the fitness center manager. First, a mobile recruitment notice was distributed to members. Applicants were screened for eligibility, and written consent was obtained from those selected before data collection began. The sample size was determined by theoretical saturation, defined as the point at which no new concepts emerged. Data collection and analysis proceeded simultaneously through in-depth interviews and participant observation, with theoretical sampling guided by the constant comparison method. Initially, interviews were conducted with nine middle-aged women who met the criteria and reported discomfort due to menopausal symptoms while participating in physical activity. After preliminary analysis, two additional participants were selected who had experienced significant personal life changes through physical activity, in order to clarify emerging concepts. Subsequently, in comparing and analyzing participant characteristics, two more women with experiences of deep immersion in physical activity were added. Theoretical saturation was judged to have been reached with a total of 13 participants. Therefore, the final study sample consisted of 13 middle-aged women.

### Data collection

Data collection was conducted over a 10-month period from September 2022 to June 2023. The primary method involved in-depth interviews, complemented by direct participant observation during physical activity programs to strengthen the accuracy of content analysis. Semi-structured interview protocols guided the process, and a standardized procedure was applied for all participants. To incorporate prior analysis into subsequent sessions, an interval of at least one week was maintained between interviews. Each participant underwent one main interview lasting 90 to 150 minutes (average 120 minutes), conducted face-to-face in a private and quiet café or, at the request of two participants (participants 2 and 4), in an empty office within the center. Following these initial sessions, additional follow-up interviews were conducted as needed—two participants participated in one supplementary 10-minute interview each, either by telephone or immediately after physical activity at the center. Throughout all interviews, field notes were used to record atmosphere, attitudes, and postures, which were incorporated into data analysis. Key questions included: “What was the most difficult aspect of the menopausal transition, and what is your current situation?” “What physical activity are you currently practicing, and what motivated you to start?” “How have those around you reacted to your physical activity?” “What helped you practice physical activity?” “What emotions did you feel while performing physical activity?” “What changes have you experienced compared to before starting physical activity?” and “How has the experience of physical activity impacted your life?” Participants were informed about their rights, confidentiality, and data protection policies before participation. With consent, interviews were audio-recorded using a portable recorder and transcribed by the author. Pseudonyms were used during transcription to ensure anonymity. Draft transcripts were sent to each participant individually via mobile file to verify accuracy, and no revisions were required. As compensation, all participants received 20,000 won in cash as a transportation allowance after completing their interviews.

### Data analysis

In this study, data were analyzed using Strauss and Corbin’s [25] grounded theory method. Analysis was performed concurrently with data collection, with ongoing efforts to maintain theoretical sensitivity. Theoretical sampling, constant comparison, memos, and diagrams were utilized, and analysis followed three stages of coding. In the first stage, open coding, the collected data were comparatively analyzed to derive concepts and categories. Interview transcripts were examined line by line, with meaningful statements underlined and concepts derived through continuous questioning and comparison. Derived concepts were compared in multiple directions, applying theoretical comparison to develop category attributes and dimensions. Similar concepts were integrated into subcategories, which were further abstracted into higher-level categories. In the second stage, axial coding, the categories identified in open coding were connected using a paradigm model to analyze their structure. The central phenomenon was identified by repeatedly questioning participants’ main concerns. Causal and contextual conditions leading to the central phenomenon were examined, as well as the strategies participants used to resolve it, the intervening conditions influencing those strategies, and the resulting consequences. The process model was further elaborated by analyzing the strategic stages over time under contextual and intervening conditions. In the third stage, selective coding, the overall situation was described in narrative form, and the core category—the central theme of the study—was derived through ongoing questioning and reflection. Relationships between categories were identified in relation to the core category. Finally, after examining both micro and macro perspectives, a situational model was constructed to integrate all elements into a comprehensive explanation.

### Rigor of the study

This study endeavored to ensure the rigor of qualitative research in accordance with the criteria proposed by Guba and Lincoln [27]. To secure truth value, the authors directly participated in physical activity programs with the participants, closely observed them, conducted in-depth and brief interviews, utilized field notes, and applied a member-check process. To establish applicability, sufficient data were collected until theoretical saturation was reached, and two middle-aged women who were consistently engaged in physical activity—selected arbitrarily from the authors’ acquaintances—were asked to reflect on their experiences, thereby confirming whether the meaning of the findings could be conveyed beyond the immediate study context. To maintain consistency, the study was conducted within the established framework of data collection and analysis, with all processes described in detail to allow replication by other researchers; in addition, the results were reviewed by a doctor of nursing and a physical activity expert to check data consistency. Finally, to ensure neutrality, the researchers engaged in continuous bracketing and reflective practice, avoided leading questions during interviews, and recorded the interview content verbatim in order to capture and understand participants’ experiences from their own perspectives.

### Preparation of researchers

The first author completed doctoral coursework in qualitative research methods, nursing theory development, and women’s health nursing, and gained experience presenting and demonstrating physical activity programs through a nursing intervention class. Training to develop qualitative research skills included conducting a preliminary study using in-depth interviews and grounded theory. The author also published a qualitative study on the physical activity experiences of perimenopausal women in a nursing journal. During this study, the author participated in physical activity programs alongside participants from beginning to end, engaging in the same activities to ensure accurate collection and analysis of research data.

## Results

### Characteristics of study participants

The total number of participants in this study was 13, with an age range of 50–59 years and a mean age of 54.5 years (Table 1). Menopausal status was classified according to the menopause stages proposed by SWAN (the Study of Women’s Health Across the Nation) [1]: 3 participants were in early perimenopause, exhibiting menstrual cycle changes; three participants were in late perimenopause, experiencing amenorrhea for 3–11 months; and seven participants were in postmenopause, with amenorrhea for 12 months or longer. Their mean age at menopause was 50.7 years. Participants engaged in physical activity at least three times per week, each session lasting 50 minutes or more. The average duration of maintaining physical activity was 3.4 years, ranging from 4 months to 10 years. For participants involved in two or more activities, the calculation was based on the longest-maintained activity. Most participants engaged in physical activity primarily through group exercise programs, though they were also free to participate individually in equipment-based activities within the center, without restrictions on time or frequency. Regarding marital status, one participant was single, two were divorced, and the remainder were married. Among the 13 women, one had diabetes, one had a breast cyst, and two had osteoporosis.

### Categorical analysis of physical activity experiences during the menopause transition

**Figure 1.**
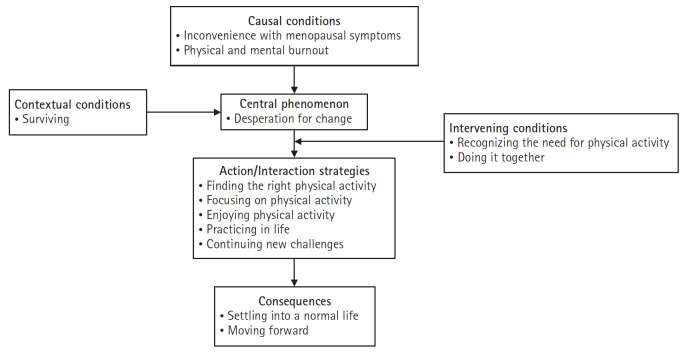
Paradigm model of middle-aged women’s experiences of physical activity during the menopausal transition.

Through open coding, 74 concepts were derived from the data and subsequently abstracted into 28 subcategories and 13 categories (Table 2). The core category was identified as “continuing my new daily life while improving my body and mind.” During axial coding, relationships among categories were examined, and a paradigm model was constructed (Figure 1).

#### Causal conditions

##### 1) Inconvenience with menopausal symptoms

In this study, “inconvenience with menopausal symptoms” refers to an unstable psychological state marked by suffering and distress caused by the rapid bodily changes associated with menopause. Participants frequently reported embarrassment and discomfort in interpersonal interactions due to fluctuating emotions, frustration over sudden weight gain and abdominal fat, and unease at perceiving themselves as aging quickly. This category was abstracted from the subcategories “frequently suffering from menopausal symptoms” and “upset about rapid changes in my body.”

“It was extremely painful because I kept getting hot and cold constantly, and I felt unable to control my body (Participant 2).”

“I suddenly found that I was older, and on top of that, I suddenly gained weight, so I felt uncomfortable and really struggled (Participant 7).”

##### 2) Physical and mental burnout

In this study, “physical and mental burnout” refers to a condition in which participants felt exhausted both physically and mentally, depleted of vitality. They described poor sleep quality leading to fatigue, ongoing instability in life that made them feel insignificant, and a gradual loss of motivation to live. This category was abstracted from the subcategories “feeling exhausted and depleted,” “wandering day and night,” and “a lack of desire for life.”

“My health status turned out to be very poor, and in the test results, my physical function was right at the bottom (Participant 2).”

“On days I couldn't sleep well, I was dazed all morning and felt tired all day (Participant 4).”

“I feel lethargic, exhausted even while lying down all day, and have lost the desire to live (Participant 12).”

#### Contextual conditions

##### 1) Surviving

In this study, “surviving (continuing the thread of life)” refers to maintaining daily life despite feeling both physically and mentally exhausted. Participants reported making efforts to return to ordinary routines while experiencing fatigue and depletion and described practicing physical activity with all their strength in order to survive. This category was abstracted from the subcategories “struggling to survive” and “continuing the rest of my life.”

“I struggled with all my might to live because I thought something terrible would happen if I delayed any further (Participant 6).”

“It’s because I want to live, and to live the rest of my life without being sicker, so I exercise (Participant 11).”

#### Central phenomenon

##### 1) Desperation for change

In this study, “desperation for change” refers to an earnest desire to escape from current suffering and to restore a stable, secure life. Participants expressed urgency in wanting to break free from pain as quickly as possible, unwillingness to grow old without any means of coping, and an earnest desire to return to stability. This category was abstracted from the subcategories “a wish for a better life,” “wanting to escape from pain,” and “not wanting to grow old like this.”

“Please, I wish life would be even a little better than it is now (Participant 3).”

“I really wanted to escape from the pain that seemed endless, day and night (Participant 9).”

“I didn’t want my appearance to be ruined. I didn’t want to grow old in this messed-up state (Participant 13).”

#### Intervening conditions

##### 1) Recognizing the need for physical activity

Participants recognized the necessity of physical activity as a way to survive the suffering that accompanied menopause. They expressed a belief in the health benefits of physical activity and considered it an indispensable element of life. This category was abstracted from the subcategories “awareness of the necessity of physical activity” and “belief in the benefits of physical activity.”

“It seems that it is difficult to live without exercise as you get older, even if you can manage to do so when you are young (Participant 7).”

“It seems that people who have exercised since their youth pass through menopause more easily (Participant 8).”

##### 2) Doing it together

Participants stated that support and encouragement from family members and others around them helped sustain their engagement in physical activity. They also reported enjoying exercise more when performed with others and emphasized that motivation from instructors was particularly valuable. This category was abstracted from the subcategories “support from family and acquaintances” and “the joy of exercising together.”

“My daughters packed my bag in advance and came out to the entrance, saying that I absolutely must not skip physical activity (Participant 6).”

“It’s more fun with others than alone, and I think the teacher is very helpful (Participant 13).”

#### Action/interaction strategies

In this study, five action/interaction strategies were identified.

##### 1) Finding the right physical activity

Participants experimented with various forms of physical activity, carefully observing how their bodies responded, and gradually discovered which activities were best suited to them. This category was abstracted from the subcategories “checking the body’s response” and “choosing the right physical activity for me.”

“I think it is really important to exercise while carefully monitoring one’s own physical condition (Participant 6).”

“I think dance suits me well (Participant 10).”

##### 2) Focusing on physical activity

Participants devoted their full attention to maintaining physical activity. They reported willingly investing time and money, and described living with their thoughts and priorities centered on exercise. This category was abstracted from the subcategories “investing time and money” and “giving full attention.”

“Right now, I spend money and time on exercising first (Participant 1).”

“I am not really interested in anything other than exercise (Participant 13).”

##### 3) Enjoying physical activity

Participants tried to enjoy physical activity without a sense of burden. By letting go of worries and immersing themselves in the present moment, they experienced feelings of liberation and joy. This category was abstracted from the subcategories “letting go of the burdens” and “enjoying exercise as much as possible.”

“I have no burden; I just came thinking of playing and going (Participant 7).”

“Actually, these days, exercise is my joy; I live with the mindset of enjoying my daily exercise (Participant 9).”

##### 4) Practicing in life

Participants described persevering with physical activity diligently and consistently, and reported that over time, such practices naturally became part of their daily routines. This category was abstracted from the subcategories “consistently practicing in daily life” and “taking root in life.”

“I am tenacious; I always go to exercise, rain or shine (Participant 5).”

“These days, if I skip exercise, I feel restless; it seems like it has become second nature to me (Participant 7).”

##### 5) Continuing new challenges

Participants reported constantly striving to challenge themselves with new tasks while practicing physical activity and stated that they trained consistently every day. This category was abstracted from the subcategories “trying until it works” and “taking on challenges every moment.”

“I kept practicing the newly learned movements until I got used to them (Participant 4).”

“These days, I feel like I am just gritting my teeth and doing my best moment by moment (Participant 5).”

#### Consequences

##### 1) Settling into a normal life

Over time, participants gradually regained vitality in both body and mind, returning to ordinary daily life while continuing to engage in physical activity naturally. Some participants expressed satisfaction with restoring their daily routines, whereas others described physical activity as a mandatory duty. This category was abstracted from the subcategories “returning to daily life with satisfaction” and “maintaining a mandatory daily routine.”

“It is important to live each day with satisfaction while exercising like this (Participant 7).”

“After finishing the exercise, I feel relieved that I have done what I had to do today (Participant 8).”

##### 2) Moving forward

Even after regaining stability in their lives, participants did not become complacent but continued to seek new challenges. Some participants described becoming deeply immersed in physical activity, even feeling as if they were addicted, while others, more proactive, emphasized freely embracing challenges and experiencing personal growth. This category was abstracted from the subcategories “moving forward obsessively” and “advancing with a sense of initiative.”

“These days, I'm completely immersed in exercising; now, it feels like I can't live without it (Participant 1).”

“I am looking forward to and excited about my future life. I feel like I am continuously progressing (Participant 6).”

### The process of physical activity experiences during the menopausal transition

**Figure 2.**
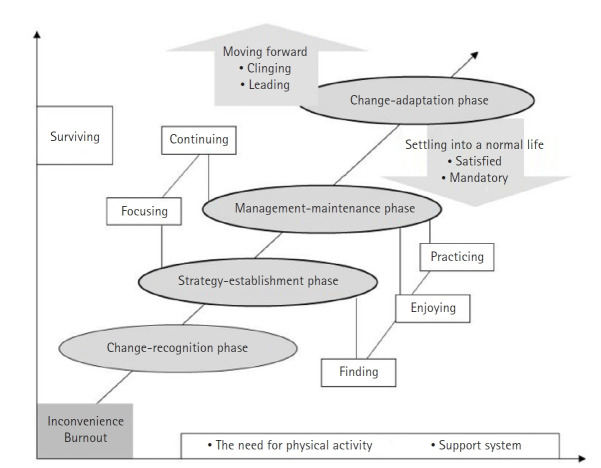
Process model of physical activity experiences during the menopausal transition.

**Figure 3.**
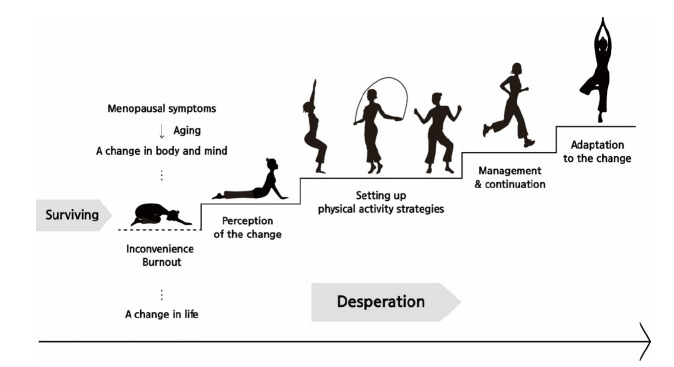
The situation model of ‘Continuing my new daily life while improving my body and mind.’

In this study, the process of participants’ physical activity experiences was delineated into four stages, which were presented in a process model (Figure 2) and further illustrated through simple physical movements in a situation model (Figure 3). The first stage, the awareness phase, involves recognizing the abrupt bodily changes associated with menopause and realizing the necessity of physical activity. The second stage, the strategy formulation phase, entails preparing countermeasures and devising strategies to overcome unstable life conditions. The third stage, the management maintenance phase, consists of applying various strategies while sustaining continuous management of daily life. The final stage, the adaptation phase, represents the point at which participants regain stability both physically and mentally through physical activity and thereby adapt to new life changes.

### Analysis of the pattern of regaining physical and mental stability to resume daily life

Based on an analysis of the recurring patterns centered on the core category, four distinct types were identified regarding how participants regained physical and mental stability to resume daily life. The first type (the settling type) is satisfaction-oriented, characterized by being generally content with the positive changes achieved through physical activity while remaining settled into daily routines. The second type (the obligation type) is practice-oriented, marked by consistent and diligent engagement in physical activity carried out with a sense of duty. The third type (the obsession type) is enthusiasm-oriented, defined by excessive preoccupation with physical activity accompanied by persistent craving. The fourth type (the growth type) is achievement-oriented, characterized by proactive engagement in challenges and continuous personal growth.

## Discussion

This study aimed to comprehensively explore the process of physical activity experiences among middle-aged women transitioning through menopause in order to assist them in adapting to this phase in a healthy manner. Participants reported experiencing negative changes in both physical and mental well-being during menopause, which accelerated aging and adversely affected their daily lives. The bodily changes associated with menopausal symptoms included weight gain and body shape changes. Some participants stated that the shock of accelerated aging and concerns overweight control served as an impetus to engage more actively in physical activity. This finding aligns with previous studies reporting that the menopausal transition is associated with weight gain and increased abdominal fat, with many women becoming overweight during menopause [28]. Prior research has also documented middle-aged women undertaking physical activity to manage weight, maintain a healthy and attractive appearance [21], or overcome midlife bodily changes [20]. However, earlier studies often emphasized motives such as curiosity, a desire for beauty [21], or nostalgia for childhood or hobbies [20], whereas this study highlights the distinct experiences of women struggling to adapt to the new changes accompanying the menopausal transition.

Participants in this study regained vitality relatively quickly as their negative self-image shifted toward a positive one. This experience supports previous research showing that an active self-image among middle-aged women enhances psychological well-being and reduces depression [29]. Nevertheless, other reports have noted that women in the menopausal transition often fail to achieve recommended levels of physical activity [16], citing lack of available time and the absence of immediate rewards as major barriers, despite the well-documented benefits. Importantly, physical activity improves both emotional and socio-psychological well-being [14]. For example, participation in an integrated health promotion program centered on group yoga, practiced at least twice a week for 12 weeks, significantly improved self-efficacy and quality of life, reduced depression, and lowered abdominal circumference and body mass index [30]. These findings suggest that physical activity can produce comprehensive improvements in both physical and mental health in a relatively short period. Moreover, previous studies have demonstrated that psychological and emotional benefits, such as stress reduction and increased happiness, can be directly experienced during physical activity [23].

Participants in this study also described a shift from negative emotions—such as awkwardness, discomfort, and fatigue—to positive ones, including satisfaction, achievement, happiness, and excitement, as they consistently engaged in physical activity. This suggests that physical activity not only facilitates emotional changes during the menopausal transition, which is already characterized by emotional instability, but also that physical activity-based interventions can support women in replacing negative emotions with healthier ones. The most exemplary group of participants was identified as the growth type, who reported experiencing transformative positive changes, often describing these as life turning points, and who were found to undergo the healthiest menopausal transition. In contrast, previous studies [31] have cited procrastination caused by low intrinsic motivation and negative perceptions of exercise as barriers to maintaining physical activity. In this study, however, the participants’ desperation for change emerged as a strong intrinsic motivator for sustaining physical activity, and their recognition of its necessity further reinforced continued participation.

In addition, this study found that new changes began at the individual level of participants once they engaged in physical activity. They reported gaining time exclusively for themselves and experiencing a sense of becoming the subjects of their own lives. This finding is consistent with previous research [2], which described middle-aged women overcoming menopausal crises and gradually becoming masters of their new lives. Similarly, in a prior study [23]—which also included some participants from this research—postmenopausal women experienced internal reflection while engaging in physical activity. Going further, the current study revealed that participants overcame difficulties during the menopausal transition by employing specific strategies, such as focusing on and immersing themselves in physical activity, and thereby naturally experienced personal growth. At the family and social/organizational levels, contextual factors also influenced participants’ menopausal transition. Whereas previous studies [31] identified family indifference as an environmental factor leading to the discontinuation of physical activity, participants in this study emphasized that family support helped them sustain their engagement. Furthermore, social and organizational programs were shown to be valuable in promoting continuity in physical activity, fostering collective solidarity, and encouraging open emotional expression. This study confirmed that support systems involving group participation and professional guidance were instrumental in helping participants adapt to life changes. This aligns with previous findings [22], which reported that women who received both physical activity and social support interventions demonstrated greater ability to cope with menopausal symptoms.

In summary, this study explored the process of physical activity experiences among middle-aged women undergoing the menopausal transition. The core phenomenon identified was a desperate need for change to regain stability in life. Participants’ experiences reflected a process of restoring exhausted bodies and minds and adapting to a renewed daily routine. Regular physical activity was shown to not only enhance physical and mental well-being but also restore vitality, enabling participants to independently overcome the challenges of the transition period. The significance of this study lies in its approach to menopause as a transitional process and in its investigation of physical activity experiences from the perspectives of women actually undergoing the transition. In doing so, it provides empirical data that can serve as a foundation for developing nursing interventions to support women during this stage. Future research is recommended to further explore the physical activity experiences of individuals who engage independently during the menopausal transition, as well as the adaptation experiences of older women who have consistently maintained physical activity since the transition period.

## Figures and Tables

**Table 1.** Characteristics of participants (N=13)

ID	Age (year)	Major symptoms of menopause	Type of physical activity	Menopause stage	Diagnosed health issues
1	54	Hot flashes and cold sweats, irritability	Yoga	Late perimenopause	
2	50	Hot flashes and cold sweats, sleep disorder	Yoga	Early perimenopause	Osteoporosis
3	52	Depression, sleep disorder	Yoga, golf	Early perimenopause	
4	50	Sleep disorder, depression	Yoga	Early perimenopause	
5	58	Sleep disorder, hot flashes, and cold sweats	Zumba, swim	Postmenopause	Diabetes
6	52	Hot flashes and cold sweats, general pain	Yoga	Postmenopause	
7	57	Sleep disorder, hot flashes, and cold sweats	Yoga	Postmenopause	
8	59	Vaginal discomfort, irritability	Aerobics	Postmenopause	
9	52	Sleep disorder, headache, hot flashes, and cold sweats	Yoga	Postmenopause	
10	56	Depression, hot flashes, and cold sweats	Belly dance, yoga	Postmenopause	Breast cyst
11	59	General pain, dry skin	Yoga, zumba, golf	Postmenopause	Osteoporosis
12	55	Vaginal discomfort, emotional change	Aerobics	Late perimenopause	
13	55	Depression, irritability	Zumba, yoga	Late perimenopause	

**Table 2.** Paradigm, categories, and subcategories of participants’ physical activity experiences

Paradigm	Categories	Subcategories
Causal conditions	Inconvenience with menopausal symptoms	Frequently suffering from menopausal symptoms
		Upset about rapid changes in my body
	Physical and mental burnout	Feeling exhausted and depleted
		Wandering day and night
		A lack of desire for life
Contextual conditions	Surviving	Struggling to survive
		Continuing the rest of my life
Central phenomenon	Desperation for change	A wish for a better life
		Wanting to escape from pain
		Not wanting to grow old like this
Intervening conditions	Recognizing the need for physical activity	Awareness of the necessity of physical activity
		Belief in the benefits of physical activity
	Doing it together	Support from family and acquaintances
		The joy of exercising together
Action/interaction strategies	Finding the right physical activity	Checking the body’s response
		Choosing the right physical activity for me
	Focusing on physical activity	Investing time and money
		Giving full attention
	Enjoying physical activity	Letting go of the burdens
		Enjoying exercise as much as possible
	Practicing in life	Consistently practicing in daily life
		Taking root in life
	Continuing new challenges	Trying until it works
		Taking on challenges every moment
Consequences	Settling into a normal life	Returning to daily life with satisfaction
		Maintaining a mandatory daily routine
	Moving forward	Moving forward obsessively
		Advancing with a sense of initiative
